# Fusion of Human Fetal Mesenchymal Stem Cells with “Degenerating” Cerebellar Neurons in Spinocerebellar Ataxia Type 1 Model Mice

**DOI:** 10.1371/journal.pone.0164202

**Published:** 2016-11-01

**Authors:** Fathul Huda, Yiping Fan, Mamiko Suzuki, Ayumu Konno, Yasunori Matsuzaki, Nobutaka Takahashi, Jerry K. Y. Chan, Hirokazu Hirai

**Affiliations:** 1 Department of Neurophysiology & Neural Repair, Gunma University Graduate School of Medicine, Maebashi, Gunma 371–8511, Japan; 2 Physiology Division, Department of Anatomy Physiology and Cell Biology, Faculty of Medicine Universitas Padjadjaran, Bandung, 40161, Indonesia; 3 Department of Reproductive Medicine, KK Women's and Children's Hospital, 229899, Singapore; 4 Experimental Fetal Medicine Group, Department of Obstetrics and Gynaecology, Yong Loo Lin School of Medicine, National University Health System, 119228, Singapore; 5 Cancer and Stem Cell Biology Program, Duke-NUS Graduate Medical School, 169857, Singapore; Faculty of Animal Sciences and Food Engineering, University of São Paulo, BRAZIL

## Abstract

Mesenchymal stem cells (MSCs) migrate to damaged tissues, where they participate in tissue repair. Human fetal MSCs (hfMSCs), compared with adult MSCs, have higher proliferation rates, a greater differentiation capacity and longer telomeres with reduced senescence. Therefore, transplantation of quality controlled hfMSCs is a promising therapeutic intervention. Previous studies have shown that intravenous or intracortical injections of MSCs result in the emergence of binucleated cerebellar Purkinje cells (PCs) containing an MSC-derived marker protein in mice, thus suggesting a fusion event. However, transdifferentiation of MSCs into PCs or transfer of a marker protein from an MSC to a PC cannot be ruled out. In this study, we unequivocally demonstrated the fusion of hfMSCs with murine PCs through a tetracycline-regulated (Tet-off) system with or without a Cre-dependent genetic inversion switch (flip-excision; FLEx). In the FLEx-Tet system, we performed intra-cerebellar injection of viral vectors expressing tetracycline transactivator (tTA) and Cre recombinase into either non-symptomatic (4-week-old) or clearly symptomatic (6–8-month-old) spinocerebellar ataxia type 1 (SCA1) mice. Then, the mice received an injection of 50,000 genetically engineered hfMSCs that expressed GFP only in the presence of Cre recombinase and tTA. We observed a significant emergence of GFP-expressing PCs and interneurons in symptomatic, but not non-symptomatic, SCA1 mice 2 weeks after the MSC injection. These results, together with the results obtained using age-matched wild-type mice, led us to conclude that hfMSCs have the potential to preferentially fuse with degenerating PCs and interneurons but not with healthy neurons.

## Introduction

Injured tissues generally release cytokines and other growth factors that induce immune responses and chemotaxis of various cell types [1,2]. Mesenchymal stem cells (MSCs) are attracted to the damaged tissues by the cytokines and exert therapeutic influence by releasing trophic factors [3–6] or transdifferentiating into the cell types in the tissue [7,8]. MSCs are readily obtained from various species and a variety of tissues (including bone marrow, adipose tissue, brain tissue and dental pulp) by using fluorescence-activated cell sorting followed by cultivation to isolate proliferative cells with adhesive properties. Whether the obtained cells are really MSCs is verified by the expression of standard mesenchymal surface antigens and trilineage differentiation into chondrocytes, adipocytes and osteoblasts. The biological properties of MSCs, which are defined only by cell surface antigens and trilineage differentiation potential, substantially differs among laboratories, depending on the species, tissue source and age of the animal from which the cells were obtained [9–11]. As a result, published results may not be reproducible if different MSCs are used [12]. Therefore, the biological quality of MSCs is critical for the success of cell therapy. Human fetal MSCs (hfMSCs) has intermediate properties between those of adult and embryonic stem cells. The advantages of hfMSCs over adult MSCs are their faster proliferative rate, higher differentiation capacity and longer telomeres with reduced senescence [13]. Therefore, high quality hfMSC lines can be expanded several log-fold and may potentially be used for many patients with various diseases, such as osteogenesis imperfecta, as allogeneic transplants [14].

Spinocerebellar ataxia type 1 (SCA1) is an inherited neurodegenerative disease caused by abnormal expansion of trinucleotide CAG repeats in the coding sequence of a causative *ATXN1* gene [15–17]. Affected individuals demonstrate neurodegeneration in multiple Central Nervous System (CNS) regions, including the cerebellum and brain stem [16]. There is no effective therapy for SCA1, and the current management approach is largely empirical and palliative.

Recently, using a murine model of SCA1 mice, we have shown that intrathecal injection of wild-type murine MSCs significantly suppresses degeneration of cerebellar Purkinje cells (PCs) and alleviates progressive ataxia in the mice [18]. More recently, we have demonstrated that injection of MSCs into the intrathecal space of SCA1 knock-in mice suppresses degeneration of motor neuron axons [19], thus suggesting the therapeutic potential of MSCs against SCA1 and potentially other neurodegenerative disorders.

Although the mechanism by which intrathecally administered MSCs ameliorate murine SCA1 pathology [18,19] remains unexplored, there are several possibilities. These include the release of trophic factors from the MSCs and the replacement of damaged neurons after their transdifferentiation into neurons. However, a different intriguing phenomenon may be the fusion of the MSCs with degenerating neurons in the cerebellum, which rescues the damaged neurons. Kemp and colleagues have previously demonstrated the presence of GFP-labeled and binucleated PCs after intravenous or intracortical administration of GFP-expressing MSCs, thus indicating that MSC fusion with PCs occurred [20,21]. However, because binucleated PCs spontaneously occur in both mice [22] and humans [23], the GFP-positive binucleated PCs found by Kemp and colleagues may have been the result of a transfer of the GFP protein or the mRNA from neighboring MSCs to binucleated PCs through gap junctions or tunneling nanotubules [24,25] without fusion. Alternatively, MSCs may be transdifferentiated into PCs, because MSCs have been shown to transdifferentiate into neuronal cells in different brain regions [26–28]. Thus, it is still unknown whether MSCs fuse with PCs. In this study, we sought to unequivocally demonstrate the fusion of hfMSCs with mouse PCs by using a genetic device that initiated GFP expression after hfMSC fusion with PCs. We found that hfMSCs fused with PCs and interneurons but only when the cells were degenerating.

## Materials and Methods

### Samples, animals and ethics

The collection of human fetal MSCs for research purposes was approved by the Domain Specific Review Board of National University Hospital, Singapore in compliance with international guidelines. Patients gave written informed consent for the use of the collected tissue. The experimental protocol, including the hfMSC injection into mouse brains, was approved through the Institutional Committee of Gunma University (No. 12–013, 12–011). SCA1 mice (B05 line) on a FVB background, which expressed mutant Ataxin-1 with an abnormally expanded polyglutamine stretch (Ataxin-1[Q82]) in PCs [29], were kindly provided by Dr. Harry T. Orr of the University of Minnesota, Minneapolis, MN, USA. SCA1 mice and wild-type mice with the same genetic background at 4 weeks and 6–8 months old were used for the experiments. The animals were housed under a 12-h light/dark cycle and given free access to food and water. All procedures for the care and treatment of animals were carried out according to the Japanese Act on the Welfare and Management of Animals, and Guidelines for Proper Conduct of Animal Experiments issued by the Science Council of Japan. All efforts were made to minimize suffering and reduce the number of animals used.

### hfMSCs isolation and culture

The hfMSCs (15-week gestation) were isolated as previously described [30,31]. Briefly, the bone marrow cells were flushed out of the femurs, and a single-cell suspension was prepared by passing the bone marrow cells through a 70 μm cell strainer (BD Biosciences, San Diego, CA). The cells were plated at a density of 10^6^/ml in Dulbecco modified Eagle’s medium (DMEM)-Glutamax (Gibco, Grand Island, NY) supplemented with 10% fetal bovine serum (Gibco) and 1% penicillin/streptomycin (Gibco) in T_75_ flasks (Nunc, Rochester, NY). hfMSCs emerged as adherent spindle-shaped cells, and the non-adherent cells were removed through medium changes every 2–3 days. hfMSCs were characterized for MSC markers (CD105^+^, CD73^+^, CD90^+^, CD45^-^, CD14^-^, CD34^-^ and HLA-DR^-^) as well as trilineage differentiation into bone, cartilage and adipose tissue [32–34]. These cells were maintained in MesenCult medium (Stemcell technologies, Cat. No. 05401, Vancouver, Canada) supplemented with hfMSC stimulatory supplements (Stemcell technologies, Cat. No. 05411) on a surface-modified polystyrene culture dish (Primaria, Corning, Tewksbury, MA) at 37°C in a 5% CO_2_, 21% O_2_ atmosphere.

### Plasmid construction

For producing adeno-associated virus serotype 9 (AAV9) vectors, we prepared 2 types of plasmids: pAAV-L7-HA-mtTA and pAAV-SynImCMV-HA-mtTA-P2A-Cre. To express transgenes specifically in PCs or various types of neurons, a PC-specific L7 promoter [35] or neuron-specific synapsin I promoter with a minimal CMV promoter sequence (SynImCMV) [36] was incorporated into the expression vector for AAV9. The mammalianized tetracycline transactivator (mtTA) cDNA [37] was kindly provided by Dr. Kenji Tanaka (Keio University). A human influenza hemagglutinin (HA) tag was placed at the amino-terminal end of mtTA (HA-mtTA) by PCR using following primers containing the HA-tag: 5´-TCCACCGGTGCCACCATGTACCCATACGATGTTCCAGATTACGCTATGGCTCGCCTGGACAAGTCCA-3´ and 5´-ATAAGAATGCGGCCGCTTATCACAGCATGTCCAGGTC-3´. The gene of HA-mtTA was inserted into the expression plasmid carrying the L7 promoter at the restriction enzyme sites for AgeI and NotI. The HA-mtTA-P2A-Cre cassette was inserted into the expression vector carrying the SynImCMV promoter at the same restriction enzyme sites. Both AAV9 vectors contained the woodchuck hepatitis post-transcriptional regulatory element (WPRE) sequence following the transgene for enhancing the expression of genes.

To produce lentiviral vectors, we constructed 3 types of plasmids: pCL20c-MSCV-GFP, pCL20c-TRE-GFP and pCL20c-TRE-FLEx-GFP. The plasmid containing the tetracycline-response element (TRE) was kindly provided by Dr. Hiroyuki Miyoshi (RIKEN BioResource Center). The plasmid containing the GFP gene in the Cre-dependent genetic switch (flip-excision; FLEx) [38] cassette was kindly provided by Dr. Tsutomu Sasaki (Gunma University). The murine stem cell virus (MSCV) promoter or TRE was inserted into the pCL20c vector [39] just upstream of a gene to be expressed. The GFP gene or FLEx-GFP was inserted into pCL20c containing the MSCV promoter or TRE at the restriction enzyme sites for AgeI and NotI. The sequences of all the plasmid constructs were confirmed by DNA sequencing using a 3130xl Genetic Analyzer (Life Technologies, Grand Island, NY).

### Production of AAV9 vectors

AAV9 vectors were produced by co-transfection of HEK293T cells with 3 plasmids as described previously: pHelper (Stratagene, La Jolla, CA, USA), pAAV2/9 (kindly provided by Dr. J. Wilson) and a pAAV expression plasmid as described [40]. The viral particles were purified using ammonium sulfate precipitation and iodixanol continuous gradient centrifugation as described previously [41]. The genomic titers of the purified AAV9 vectors were determined by real-time quantitative PCR using the following primers: 5´-CTGTTGGGCACTGACAATTC-3´ and 5´-GAAGGGACGTAGCAGAAGGA -3´ targeting the WPRE sequence. The titers of AAV9-L7-HA-mtTA and AAV9-SynImCMV-HA-mtTA-P2A-Cre were 1.2×10^12^ vg/ml and 2.9×10^14^ vg/ml, respectively. In addition to these 2 AAV9 vectors for testing hfMSC fusion with cerebellar cells, we produced one more AAV9 vector AAV9-TRE-Venus, to examine the leakage of the TRE promoter in mouse cerebella *in vivo*. The titer of AAV9-TRE-Venus was 5.7 × 10^13^ vg/ml.

### Production of lentiviral vectors and transduction of hfMSCs by the lentiviral vectors

The vesicular stomatitis virus-glycoprotein-pseudotyped lentiviral vectors were produced in HEK293T cells (Thermo Fisher Scientific, Kanagawa, Japan) as described previously [42]. The lentiviral vector plasmid DNAs were kindly provided by Dr. Nienhuis (St. Jude Children’s Research Hospital, Memphis, TN). The backbones of helper plasmids were derived from pCAGGS [43]. Briefly, four plasmids were transfected into HEK293T cells using the calcium phosphate method, and the supernatant was harvested 48 h after transfection, concentrated through ultra-centrifugation (CP80WX; Hitachi Koki, Tokyo, Japan), and resuspended in 70 μl of Dulbecco phosphate-buffered saline (−). The resultant lentiviral solution was stored at 4°C and used within 3 weeks. For transduction of hfMSCs, five hundred thousand hfMSCs were incubated with lentiviral vectors at a multiplicity of infection of 100 in a 10 ml culture medium in a 10 cm culture dish for approximately 18 h.

### Injection of hfMSCs, lentiviral vectors and AAV9 vectors into mouse brains

Cerebellar injection of AAV9 vectors and hfMSC was performed as previously described [40]. Mice were anesthetized by an intraperitoneal injection of ketamine (100 mg/kg body weight) and xylazine (16 mg/kg body weight) and mounted onto a stereotactic injection frame. For injection into the cerebellum, a burr hole was made 2.0–2.5 mm caudal from the lambda. The blunt-ended tip (33 gauge) of a Hamilton syringe attached to a micropump (UltramicroPump II; World Precision Instruments (WPI), Sarasota, FL) was inserted 1.8 mm (for 4-week-old mice) or 2 mm (for mice older than 6 months) into the cerebellar cortex. A solution containing 50,000 hfMSCs or viral vectors (10 μl) was injected at a rate of 333 nl/min. The titers of injected AAV9 vectors in the 10 μl viral solution were 2.3 × 10^9^ vg (AAV9-L7-HA-mtTA) and 1.4 × 10^10^ vg (AAV9-SynImCMV-HA-mtTA-P2A-Cre). The syringe was left in place for an additional 2 min before it was withdrawn. The scalp was then sutured, and each mouse was kept on a heating pad until recovery from the anesthesia before it was returned to a standard cage.

### Immunohistochemistry

The mice were deeply anesthetized before they were transcardially perfused with phosphate-buffered saline and a fixative containing 4% paraformaldehyde in a 0.1 M phosphate buffer. The whole brain was removed and post-fixed in the same fixative overnight. Parasagittal sections (50 μm thick) were prepared from the cerebellar vermis using a vibratome/microtome (Leica VT1000 S; Leica Microsystems, Wetzlar, Germany). Floating brain sections were immunostained in blocking solution (2% normal donkey serum and 0.4% Triton X-100 in phosphate buffer) for two days at 4°C with the primary antibodies described below. Then the slices were incubated with secondary fluorescent dye-conjugated antibodies for 4 h at room temperature.

Primary antibodies included rabbit polyclonal anti-Iba1 (1:1000; 019–19741; Wako, Osaka, Japan), rat monoclonal anti-GFP (1:1000; Clone, GF090R, Nacalai Tesque, Kyoto, Japan), rabbit monoclonal anti-GFP (1:1000; Cat. No. GFP-Rb-Af2020, Frontier Institute, Hokkaido, Japan), mouse monoclonal anti-calbindin D-28K (1:500; Cat. No. 300, Swant, Bellinzona, Switzerland) and rat monoclonal anti-HA (1:1000; Cat. No. 11867423001, Roche, Mannheim, Germany) for cerebellar sections or rat monoclonal anti-GFP (1:1000; Clone, GF090R, Nacalai) and mouse monoclonal anti-NeuN (1:1000; MAB377, Merck Millipore, Billerica, MA, USA) for cerebral sections. The secondary antibodies were Alexa Fluor 488-conjugated donkey anti-rat or anti-rabbit IgG (1:500; Life Technologies), Alexa Fluor 568-conjugated donkey anti-mouse, anti-rat IgG (1:500; Life Technologies) or anti-rabbit IgG (1:1000, Life Technologies) and Alexa Fluor 680-conjugated donkey anti-mouse IgG (1:500; Life Technologies).

### Sizes of PC and interneuron cell bodies

PC and molecular layer interneurons were immunolabeled for parvalbumin. An image of the immunoreaction to parvalbumin was obtained by manually setting an appropriate threshold, and the numbers of pixels occupied by PC soma and interneuron soma were measured with Image J software. One hundred micrometer corresponded to 261 pixels in the image that was acquired using a × 20 objective. Therefore, one pixel was equal to 0.1468 μm^2^. Thus, the sizes of PC and interneuron somata (μm^2^) were calculated by multiplying the number of pixels by 0.1468.

## Results

### Emergence of GFP-positive PCs after transplantation of hfMSCs carrying a GFP gene into the cerebellar cortex

**Fig 1:**
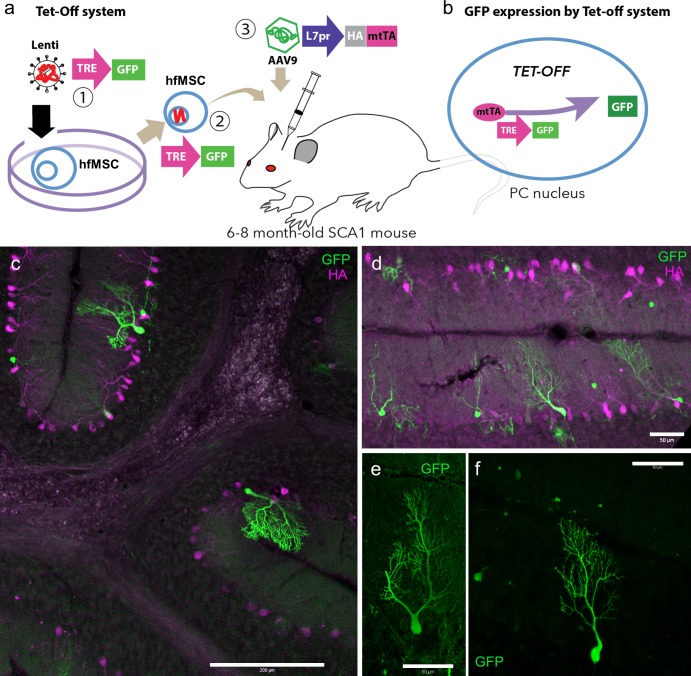
Emergence of GFP-expressing PCs in SCA1 mouse cerebella after transplantation of TRE-GFP-hfMSCs expressing GFP through the Tet-off system. (a) The TRE-GFP gene was introduced into hfMSCs through a lentivirus (1). The transduced TRE-GFP-hfMSCs were injected into the cerebella of 6–8-month-old SCA1 mice (2). Two weeks after the TRE-GFP-hfMSC injection, the SCA1 mice received an injection of AAV9-L7-HA-mtTA vectors expressing HA-tagged mtTA under the control of a PC-specific L7 promoter (3). (b) Schema depicting GFP expression in PCs expressing mtTA. TRE in the nucleus of TRE-GFP-hfMSCs initiated the transcription of the downstream GFP gene in the presence of mtTA, which was produced only in AAV9-L7-HA-mtTA-infected PCs. (c, d) Immunohistochemistry of cerebellar slices from SCA1 mice treated as shown in the schema (a). The mice were sacrificed 10 weeks after the hfMSC grafting. The slices were double immunolabeled for HA and GFP. HA-tagged mtTA (magenta) was efficiently expressed only in PCs, and some PCs co-expressed GFP (green). (e, f) Enlarged images of PCs immunostained for GFP. Scale bars, 200 μm (c) and 50 μm (d-f).

**Fig 2:**
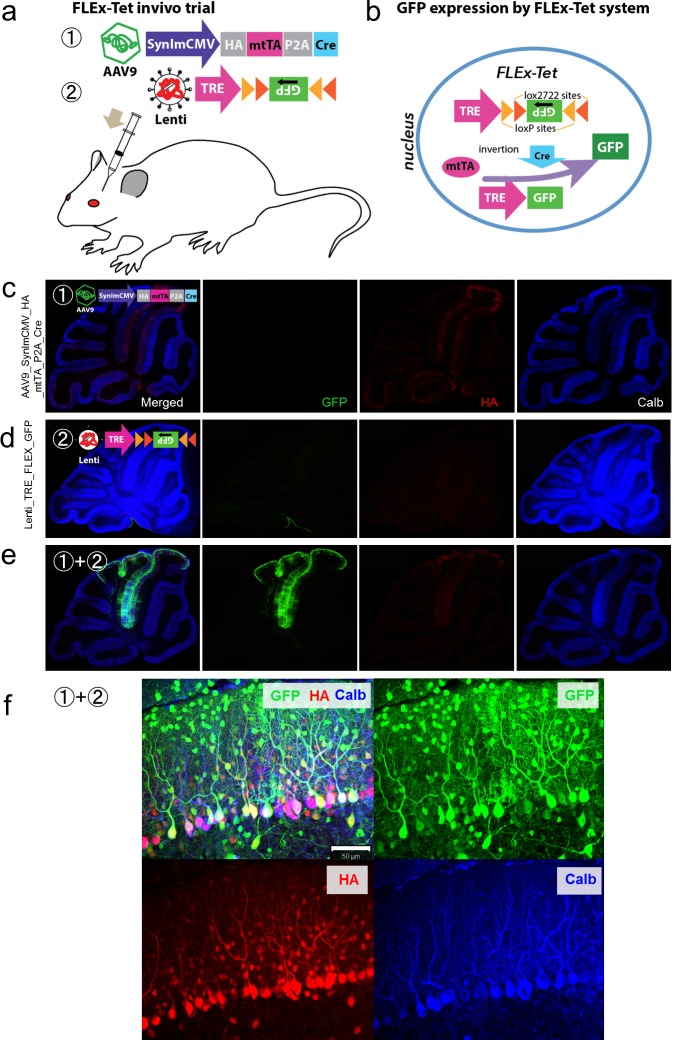
Validation of the FLEx-Tet system in mouse cerebella *in vivo*. (a) The AAV9-SynImCMV-HA-mtTA-P2A-Cre vector bicistronically expressed HA-tagged mtTA and Cre recombinase under the control of a neuron-specific SynImCMV promoter (1). P2A, which is a ‘self-cleaving’ peptide sequence, was inserted between mtTA and Cre, whereas lentiviral vectors (Lenti-TRE-FLEx-GFP) carried TRE and an inverted GFP sequence flanked by loxP and lox2272 on both sides (2). AAV9 and/or lentiviral vectors were injected into 4-week-old mice. (b) Diagram depicting the FLEx-Tet system. Cre-mediated recombination and inversion of the GFP gene permitted expression of GFP protein in the presence of mtTA. (c-e) Immunohistochemistry of the mice that received an injection of the AAV9-SynImCMV-HA-mtTA-P2A-Cre vector (c), Lenti-TRE-FLEx-GFP vector (d) or the viral mixture (e). Two weeks after the viral injection, the mice were sacrificed, and the cerebellar slices were triple immunostained for GFP, HA and calbindin. Notably, GFP was expressed only in the cerebella of mice that received an injection of the viral mixture. (f) Magnified images of GFP-expressing lobules showing expression of GFP in various types of cortical neurons, including PCs, interneurons and granule cells. Scale bar, 50 μm.

**Fig 3:**
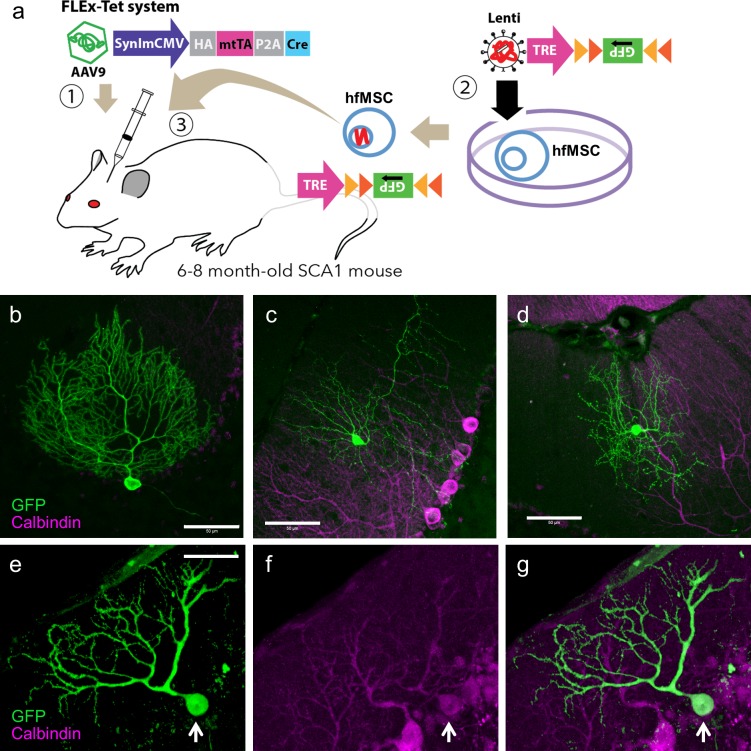
GFP expression in PCs and putative interneurons after grafting hfMSCs carrying the inverted GFP gene to the SCA1 mouse cerebellum. (a) Schema depicting the FLEx-Tet system, which permitted us to explore the fusion of hfMSCs with cerebellar neurons. An AAV9-SynImCMV-HA-mtTA-P2A-Cre vector expressing HA-tagged mtTA and Cre through a neuron-specific SynImCMV promoter was injected into the cerebella of 6–8-month-old SCA1 mice (1). A sequence with TRE and inverted GFP genes flanked by loxP and lox2272 was inserted into the genome of hfMSCs by using Lenti-TRE-FLEx-GFP vectors (2). Thus, GFP protein was never produced without Cre recombinase, and the expression increased drastically after co-provision with mtTA. The TRE-FLEx-GFP-hfMSCs (50,000 cells) were then injected into the SCA1 mice 2 weeks after injection of AAV9-SynImCMV-HA-mtTA-P2A-Cre vectors (3). (b-g) Emergence of GFP-expressing neurons in the cerebella of mice that were sacrificed 2 weeks after the TRE-FLEx-GFP-hfMSC injection. The cerebellar slices were immunolabeled for GFP and calbindin, a PC marker. GFP expression was detected in PCs (b, e-g) and cells in the molecular layer (c, d). Arrows in (e-g) indicate a GFP-expressing PCs co-immunostained for calbindin. Because we used a neuron-specific synapsin I promoter with a minimal CMV sequence, Cre and mtTA should have been expressed specifically in neurons, and thus the GFP-expressing cells in the molecular layer (c, d) were presumed to be basket cells (c) and stellate cells (d). Scale bar, 50 μm.

MSCs secrete a variety of growth factors that have both paracrine and autocrine activities in the damaged brain [25,44]. In contrast, an intriguing possibility (supported by limited evidence) is the fusion of the grafted MSCs with host neurons. Therefore, we examined the fusogenic potential of hfMSCs in the cerebellum and the frequency at which they fused with cerebellar neurons. To obtain robust GFP expression, we used a tetracycline (Tet)-controlled expression system. TRE and GFP sequences were introduced into the hfMSC genome by using lentiviral vectors carrying TRE-GFP (Fig 1A-1). The viral infection had no obvious influence on hfMSC viability in terms of growth rate (data not shown). Fifty thousand TRE-GFP-bearing hfMSCs (TRE-GFP-hfMSC) were injected into the cerebellar cortex of symptomatic 6–8-month-old SCA1 mice (Fig 1A-2). Two weeks after the TRE-GFP-hfMSC transplantation, the mice received an injection of AAV9-L7-HA-mtTA vectors expressing a mtTA tagged at the N-terminus with HA under the control of a PC-specific L7 promoter (Fig 1A-3), which restricted expression of mtTA to PCs. Thus, only PCs harboring the TRE-GFP construct were expected to express GFP after expression of mtTA (Fig 1B). Ten weeks after the TRE-GFP-hfMSC injection (8 weeks after AAV9-L7-HA-mtTA injection), the mice were sacrificed, and cerebellar slices were double immunostained for GFP and HA. We found highly efficient expression of HA-mtTA exclusively in PCs (magenta, Fig 1C and 1D) together with numerous GFP-labeled PCs (green, Fig 1C–1F). This result suggests that fusion of TRE-GFP-hfMSCs occurred with AAV9-L7-HA-mtTA-infected PCs and/or the transdifferentiation of TRE-GFP-hfMSCs into PCs.

### Emergence of GFP-positive cells in the molecular layer of the cerebellum

Because TRE has leaky promoter activity even in the absence of mtTA [45], injected TRE-GFP-hfMSCs might be gradually labeled with GFP. We assessed this possibility by injecting AAV9-TRE-Venus into mouse cerebella. Two weeks after the AAV9-TRE-Venus injection, we found a few Venus-expressing cells in the cerebellar sections, although mtTA had not been applied. When AAV9-TRE-Venus-injected mice were sacrificed 4 or 10 weeks after the viral injection, we found a time-dependent increase in Venus fluorescence in the cerebellar sections (data not shown). These results suggest that a moderate amount of GFP protein, as detected with immunohistochemistry, was produced and gradually accumulated in TRE-GFP-hfMSCs over time.

To completely eliminate the expression of GFP in TRE-GFP-hfMSCs that was generated by leaky TRE activity, we switched to using a FLEx system [46]. GFP downstream of TRE was inserted in reverse with loxP and lox2272 flanking both sides (Fig 2A-2). Thus, TRE-FLEx-GFP-hfMSCs expressed GFP only when Cre-mediated recombination reversed the orientation of the GFP gene, and GFP expression was augmented by the supply of mtTA (FLEx-Tet system, Fig 2B). Cre recombinase and mtTA were delivered to the cerebellum through AAV9-SynImCMV-HA-mtTA-P2A-Cre vectors, which expressed mtTA and Cre bicistronically through a synapsin I promoter with a minimal CMV sequence (SynImCMV) (Fig 2A-1). To express mtTA and Cre in all neuronal cell types in the cerebellum, we used a neuron-specific SynImCMV promoter [36] instead of a PC-specific promoter because we had been unable to detect the fusion of hfMSCs with other neuronal cell types.

To validate this FLEx-Tet system, we injected AAV9-SynImCMV-HA-mtTA-P2A-Cre vectors and lentiviral vectors carrying TRE-FLEx-GFP (Lenti-TRE-FLEx-GFP) separately or simultaneously into mouse cerebella. No GFP-expressing cells were found in the cerebella of mice that received an injection of either AAV9-SynImCMV-HA-mtTA-P2A-Cre vectors or Lenti-TRE-FLEx-GFP alone (Fig 2C and 2D), although mtTA expression was identified by immunostaining in the cerebella treated with AAV9-SynImCMV-HA-mtTA-P2A-Cre vectors (Fig 2C). GFP expression was observed only in the cerebella of mice that received an injection of both AAV9-SynImCMV-HA-mtTA-P2A-Cre and Lenti-TRE-FLEx-GFP vectors (Fig 2E). We observed GFP expression in different neuronal cell types that were morphologically similar to PCs, interneurons and granule cells (Fig 2F). These findings indicated that the combination of Tet-controlled technology with the FLEx system worked in the cerebellar neurons.

After the validation of the FLEx-Tet system, we injected the AAV9-SynImCMV-HA-mtTA-P2A-Cre vectors (Fig 3A-1) into the cerebella of 6–8-month-old SCA1 mice and then injected 50,000 TRE-FLEx-GFP-hfMSCs two weeks later (Fig 3A-2,3). Two weeks after hfMSC transplantation (4 weeks after AAV vector injection), we found GFP-expressing PCs (Fig 3B) and two morphologically distinct cells in the molecular layer (Fig 3C and 3D). The GFP-expressing PCs co-immunostained for calbindin (Fig 3E–3G). Since we have previously found that 97% of transduced cells in the cerebellum with a neuron-specific promoter (SynIminCMV) were theoretically neurons [36], the morphological similarity of GFP-labeled cells in the molecular layer indicated that they could be basket cells (Fig 3C) or stellate cells (Fig 3D). These results strongly suggest that hfMSCs transplanted into the cerebellar cortex fuse with PCs, and presumably interneurons as well, within two weeks of transplantation.

### Fusion frequency of hfMSCs and cerebellar neurons

We next examined the emerging frequency of GFP-labeled cells in 6–8-month-old SCA1 mice. Injection of 50,000 hfMSCs into the cerebellum resulted in the appearance of GFP-positive cells close to the injection site. In the Tet-off system (Fig 1), we extensively searched 4 SCA1 mice at 6–8 months of age (Table 1) and identified 92 GFP-positive cells from 4 mice (Table 2). In the FLEx-Tet system, we examined 12 SCA1 mice at 6–8 months of age (Table 1) and found 33 GFP-positive cells from 2 mice that were examined 2 weeks after the hfMSC injection (Table 2), whereas 33 GFP-positive cells were identified in 3 mice that were examined 23–25 weeks after the hfMSC injection (Table 2). These results confirm that fusion of hfMSCs with cerebellar neurons occurs within two weeks of transplantation, although a constant flux of continued MSC fusion and inactivation of fused MSCs from either the donor or recipient cell nuclei cannot be excluded.

**Table 1:** Summary of hfMSC transplantation into the cerebellum of SCA1 mice.

Genotype	Injection age	No. of mice examined	Incubation period (weeks)	No. of mice that yielded GFP(+) cells	No. of mice (FLEx-Tet/Tet-off)
SCA1	4 weeks old	12	4–21	0	4 / 8
	6–8 months old	16	2–26	9	12 / 4
WT	4 weeks old	11	10	1	0 / 11
	6–8 months old	10	2–5	0	0 / 10

**Table 2:** Summary of mice that expressed GFP-labeled cells in the cerebellum.

Genotype	Injection age	Mouse No.	Incubation period (weeks)	No. of GFP (+) cells	System
WT	4 weeks old	1	10	1	Tet-off
SCA1	6–8 months old	1	2	30	FLEx-Tet
		2	2	3	FLEx-Tet
		3	2	3	Tet-off
		4	2	2	Tet-off
		5	10	44	Tet-off
		6	10	43	Tet-off
		7	23	16	FLEx-Tet
		8	25	8	FLEx-Tet
		9	25	9	FLEx-Tet

To clarify whether hfMSCs have the potential to fuse with non-degenerating neurons, we performed similar experiments using asymptomatic 4-week-old SCA1 mice in which histo-morphological degeneration in the cerebellum had not begun. Four mice that were designed to express GFP through the FLEx-Tet system (Fig 3A) and 8 mice that were designed to express GFP through the Tet-off system (Fig 1A and 1B) were examined, and no GFP-labeled cells were detected (Table 1). To confirm our hypothesis, we performed similar Tet-off experiments using 4-week-old and 6-month-old wild-type mice (11 and 10 mice, respectively). In total 21 wild-type mice examined, we found just one GFP-expressing cell in the cerebellum of a 4-week-old wild-type mouse (Table 1).

**Fig 4:**
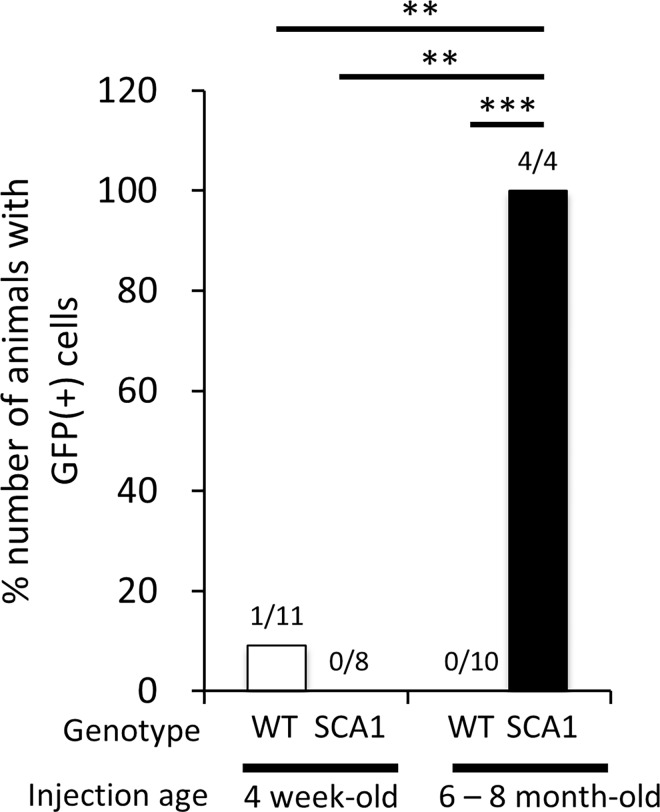
A summary graph showing the percent number of mice that yielded GFP-positive cells after the hfMSC grafting to the cerebellum. Four-week-old and 6–8-month-old wild-type (WT) and SCA1 mice that were treated with the Tet-off system (Tables 1 and 2) were analyzed. All mice were incubated more than 2 weeks and, therefore, those that did not show GFP (+) cells at the time of microscopic observation were regarded as fusion-negative mice. Number on each bar shows number of mice with GFP (+) cells / that of mice examined. Fisher’s exact test showed significantly higher emergence of GFP-positive cells in 6–8-month-old SCA1 mice than 4-week-old and 6–8-month-old wild-type mice and 4-week-old SCA1 mice. **p<0.01, ***p<0.001.

We next performed the statistical analysis. Here, we used 2 different systems, Tet-off system and FLEx-Tet system. In the Tet-off system, since GFP was expressed only in cells virally expressing mtTA, the GFP expression ratio was affected by AAV9-mediated transduction and efficiency, thus suggesting that the frequency of GFP-labeled cells emerging from the Tet-off system was lower than the actual fusion rate of hfMSCs with cerebellar neurons. In the FLEx-Tet system, the GFP expression frequency depends on the Cre-mediated recombination rate in addition to AAV9-mediated transduction efficiency of neurons. Thus, the emergence frequency of GFP-positive cells in the FLEx-Tet system is lower than the frequency in the Tet-off system. Therefore, for the proper comparison, we used only the results obtained by the Tet-off system and focused solely on the emergence, not the frequency, of the fused cells for the statistics. Another point that we need to consider would be the variable incubation period after the hfMSC injection. As for this point, since the hfMSC fusion with cerebellar neurons was observed at 2 weeks post-injection, mice that did not yield GFP-positive cells over 2 weeks after the hfMSC injection were regarded as fusion-negative mice. As the populations for the statistics were small, we used Fisher’s exact test rather than χ^2^ test. The analysis revealed significantly higher (and almost exclusive) emergence of GFP-positive cells in 6–8-month-old SCA1 mice, compared with 4-week-old (**p<0.01) and 6–8-month-old (***p<0.001) wild-type mice and 4-week-old SCA1 mice (**p<0.01) (Fig 4).

### Interneurons in the molecular layer of SCA1 mice demonstrate non-cell autonomous degeneration

**Fig 5:**
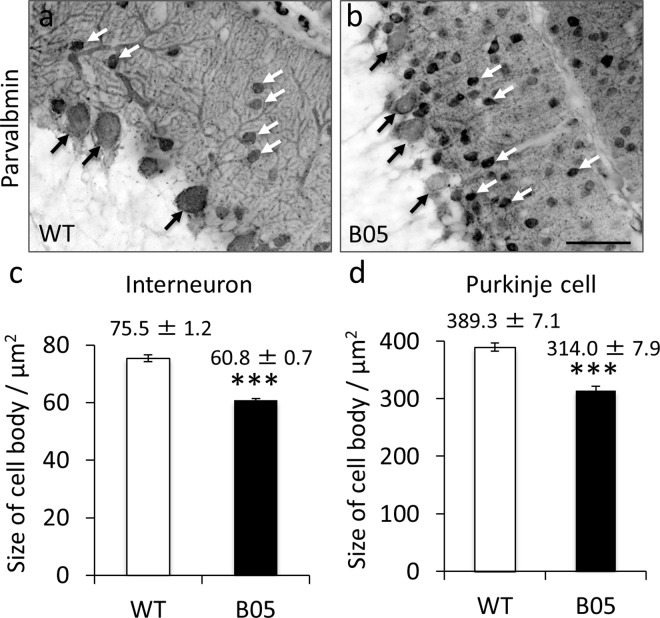
Degeneration of PC and molecular layer interneurons. (a, b) Fluorescence images of the cerebellar cortex immunostained for parvalbumin from a 6-month-old wild-type mouse (WT) (a) and an age-matched SCA1 mouse (B05) (b). Black and white arrows show examples of PCs and interneurons, respectively. Scale bar, 50 μm. (c, d) Graphs showing the size of cell bodies of interneurons (WT; n = 110 cells from 3 mice, B05; n = 140 cells from 4 mice) (c) and of PCs (WT; n = 81 PCs from 3 mice, B05; n = 80 PCs from 4 mice) (d). Asterisks indicate a statistically significant difference between the wild-type mice and SCA1 mice; ***p<0.001 by unpaired *t*-test.

If hfMSCs are more likely to fuse with degenerating neurons, the molecular layer interneurons of SCA1 mice should be degenerated because GFP-expressing cells, which were inferred to be interneurons, were observed in the molecular layer (Fig 3C and 3D). However, the SCA1 mice (B05 line), which have transgene expression driven by the PC-specific L7 promoter, express aberrant Ataxin-1[Q82] protein only in PCs [29] and only PCs were affected. Of course, because cells around PCs tightly associate with and may be subject to trophic influence from PCs, it would not be surprising if the molecular layer interneurons in the symptomatic SCA1 mice were degenerated. Therefore, we examined the degeneration of the molecular layer interneurons by assessing the size of the interneuron soma, because degenerated neurons have shrunken cell bodies [47]. The size of PC soma in 6-month-old SCA1 mice was significantly smaller than that in age-matched wild-type mice (***p<0.001 by unpaired *t*-test) (Fig 5A, 5B and 5D). Similarly, the size of the interneuron soma in 6-month-old SCA1 mice was much smaller than in age-matched wild-type mice (***p<0.001 by unpaired *t*-test) (Fig 5A–5C). This outcome suggests that non-cell autonomous degeneration of the molecular layer interneurons occurred in 6-month-old SCA1 mice, in agreement with our notion that degenerating neurons preferentially fuse with hfMSCs.

## Discussion

In this study, we used Tet-off and FLEx-Tet systems to demonstrate that hfMSCs injected into the cerebella of symptomatic SCA1 mice fuse with cerebellar neurons, including PCs and molecular layer interneurons, whereas no fusion occurs in the cerebella of non-symptomatic SCA1 mice or in the cerebella of young and elderly wild-type mice. Moreover, we observed fusion occurring within 2 weeks after the hfMSC transplantation into the cerebella, which is far earlier than the fusion resulting from intravenous injection of bone marrow derived cells (BMDCs) (at least 24 weeks) [48].

MSCs are of great clinical interest because of their self-renewal capacity and their differentiation and therapeutic potential. MSCs from human fetuses have considerable advantages over adult MSCs, including their higher proliferative rate, greater differentiation potential and longer telomeres [13]; these characteristics might allow prolonged *ex vivo* expansion to obtain sufficient cell numbers for treating a large number of patients. It has been reported that grafting hfMSCs into developing murine brains leads to integration with the brain tissue, but the proportion of hfMSCs that transdifferentiate into oligodendrocytes remains very low. However, the proportion can be increased significantly by exposing the cells to differentiation medium *in vitro* before transplantation [49]. Thus, the behavior of hfMSCs *in vitro* is significantly different from the *in vivo* brain tissue and depended on the differences in pretreatments and environments in which the cells are grafted. It is important to gather data on various hfMSC behaviors to facilitate future utilization of hfMSCs as research and therapeutic resources.

The fusion of peripheral stem cells with PCs has mainly been studied using BMDCs [22,48,50–53]. For example, Alvarez-Dolado et al. have convincingly demonstrated fusion of BMDCs with PCs through transplantation of BMDCs expressing Cre and GFP into the peripheral circulation of lethally irradiated R26R transgenic mice expressing LacZ after Cre/lox recombination, although the frequency was very low [48,50]. Later, Magrassi et al. [22] showed that the number of PCs fusing with BMDCs in mice increases by 10 times when PCs are pharmacologically degenerated, thus suggesting the increased fusogenic potential of degenerating PCs. As for MSCs, Jones et al. have injected bone marrow-derived MSCs from young adult GFP transgenic mice directly into the cerebellar parenchyma of newborn *Lurcher* mutant mice exhibiting PC degeneration and eventual ataxia from as early as the first postnatal week, and they found a small number of GFP-positive PCs in the cerebella of 2-month-old *Lurcher* mice [54]. Although those PCs may have been labeled with GFP, there is a possibility that the GFP-positive PCs were a consequence of a transfer of GFP protein or the mRNA from GFP-expressing MSCs to PCs [25,55,56]. The presence of two nuclei in one PC does not necessarily prove fusion of an injected MSC with the PC because ~5% of PCs are binucleated in the cerebella of 18-month-old healthy mice, and the frequency increases markedly in compromised cerebella [22]. In addition, the number of binucleated PCs was 100-fold more than the number of GFP-expressing fused PCs in aged chimeric mice that received a bone marrow transplant [22], thus suggesting that BDMC fusion is not the only way to generate a binucleated PC. Therefore, it is still controversial whether MSCs fuse with PCs, and we sought to demonstrate fusion of MSCs with PCs in this study.

In the initial experiments, we used a Tet-off system in which hfMSCs carrying TRE-GFP were injected into SCA1 mice. Theoretically, the TRE promoter should be silent in the absence of tTA, and no GFP was produced in hfMSCs even when they were transdifferentiated to PCs. However, the TRE promoter did have leaky promoter activity, and therefore it was possible that the observed GFP-positive PCs were a consequence of transdifferentiation from hfMSCs. With respect to the morphological aspects, such a rapid and extensive differentiation of PC dendrites (Fig 1C–1F) occurring only 10 weeks after grafting seemed unlikely. In the second round of experiments, we used a FLEx-Tet system to eliminate GFP expression from the leaky TRE promoter activity. In this system, the hfMSCs possessed an inverted GFP sequence downstream of the TRE promoter. Although the chance was almost negligible, possible transfer of mRNAs and/or proteins of both Cre and mtTA from virally transduced host (mouse) neurons to human PCs transdifferentiated from hfMSCs might explain the emergence of GFP-positive PCs without fusion. Nevertheless, it would be almost impossible to extend and form the mature PC dendrites presented in Fig 3B just 2 weeks after transplantation. Therefore, we concluded that GFP-labeled neurons were produced by hfMSC fusion with host neurons.

The pathophysiological significance of the fusion of hfMSCs with cerebellar neurons has not been clarified yet. Because the fusion frequency increased drastically in degenerating neurons in hfMSCs (in this study) as well as BMDCs [22], fusion might have a therapeutic benefit in degenerating neurons, which was suggested in previous reports [3,18,57,58]. It has been shown that the nuclei of BMDCs derived from rats produced rat proteins specific to PCs in mouse cerebella [59], which indicates that the nuclei of donor cells were transdifferentiated to nuclei with a host cell lineage and initiated transcription of PC-specific genes after cell fusion. A PC nucleus of a SCA1 mouse possessed a mutant Ataxin-1[Q82] gene and produced toxic mutant Ataxin-1 protein. Therefore, if the host nucleus carrying the mutant gene was inactivated and replaced with a donor nucleus that has a corresponding healthy gene, the fusion may result in termination of mutant protein production and therefore rescue neurons from degeneration.

## Conclusions

Using the FLEx-Tet system, we distinctly showed the fusion of hfMSCs with PCs and interneurons when hfMSCs were grafted into the cerebella of SCA1 mice. The hfMSCs fused exclusively with degenerated neurons within 2 weeks after transplantation. If the fusion rate could be substantially elevated, the hfMSC fusion with the degenerating neurons may have significant potential to rescue the cerebellum from degeneration and should therefore be exploited as a therapeutic intervention.

## Abbreviations

AAV9: adeno-associated virus serotype 9
FLEx: flip-excision
HA: hemagglutinin
hfMSC: human fetal mesenchymal stem cell
mtTA: mammalianized tetracycline transactivator
PCs: Purkinje cells
SCA1: Spinocerebellar ataxia type 1
SynImCMV: synapsin I promoter with minimal CMV sequence
Tet: tetracycline
TRE: tetracycline-response element.
